# MRI-Derived Apparent Diffusion Coefficient of Peri-Prostatic Adipose Tissue Is a Potential Determinant of Prostate Cancer Aggressiveness in Preoperative Setting: A Preliminary Report

**DOI:** 10.3390/ijerph192315996

**Published:** 2022-11-30

**Authors:** Alessandro Tafuri, Andrea Panunzio, Federico Greco, Antonella Maglietta, Francesco De Carlo, Federica Di Cosmo, Elia Luperto, Mino Rizzo, Arturo Cavaliere, Rita De Mitri, Federico Zacheo, Marco Baviello, Alessandra Cimino, Marco Pisino, Luca Giordano, Caterina Accettura, Antonio Benito Porcaro, Alessandro Antonelli, Maria Angela Cerruto, Elisa Ciurlia, Silvana Leo, Luigi Giuseppe Quarta, Vincenzo Pagliarulo

**Affiliations:** 1 Department of Urology, “Vito Fazzi” Hospital, Piazza Filippo Muratore, 1, 73100 Lecce, Italy; 2 Department of Urology, University of Verona, Azienda Ospedaliera Universitaria Integrata di Verona, 37126 Verona, Italy; 3 U.O.C. Diagnostica per Immagini Territoriale Aziendale, Cittadella della Salute Azienda Sanitaria Locale di Lecce, Piazza Filippo Bottazzi, 73100 Lecce, Italy; 4 Department of Pathology, “Vito Fazzi” Hospital, 73100 Lecce, Italy; 5 Department of Nuclear Medicine, “Vito Fazzi” Hospital, 73100 Lecce, Italy; 6 Department of Oncology, “Vito Fazzi” Hospital, Piazza Filippo Muratore 1, 73100 Lecce, Italy; 7 Department of Radiology, “Vito Fazzi” Hospital, 73100 Lecce, Italy; 8 Department of Radiation Therapy, “Vito Fazzi” Hospital, 73100 Lecce, Italy

**Keywords:** prostate biopsy, prostate cancer, MRI, ADC, periprostatic adipose tissue

## Abstract

Background: The aim of this study was to test the association between periprostatic adipose tissue (PPAT)—apparent diffusion coefficient (ADC) value recorded at multiparametric magnetic resonance imaging (mpMRI) and determinants of prostate cancer (PCa) aggressiveness in the preoperative setting. Methods: Data from 219 consecutive patients undergoing prostate biopsy (PBx) for suspicion of PCa, between January 2020 and June 2022, at our institution were retrospectively evaluated. Only patients who had mpMRI performed before PBx were included. The distribution of demographics and clinical features among PPAT-ADC values up to vs. above the median was studied using both parametric and non-parametric tests, according to variables. Linear and logistic regression models tested the association between PPAT-ADC values and determinants of PCa aggressiveness and the presence of intermediate-high risk PCa, respectively. Results: Of 132 included patients, 76 (58%) had PCa. Median PPAT-ADC was 876 (interquartile range: 654 − 1112) × 10^−6^ mm^2^/s. Patients with PPAT-ADC up to the median had a higher rate of PIRADS (Prostate Imaging—Reporting and Data System) 5 lesions (41% vs. 23%, *p* = 0.032), a higher percentage of PBx positive cores (25% vs. 6%, *p* = 0.049) and more frequently harbored ISUP (International Society of Urological Pathology) > 1 PCa (50% vs. 28%, *p* = 0.048). At univariable linear regression analyses, prostate-specific antigen (PSA), PSA density, PIRADS 5, and percentage of PBx positive cores were associated with lower PPAT-ADC values. PPAT-ADC up to the median was an independent predictor for intermediate-high risk PCa (odds ratio: 3.24, 95%CI: 1.17–9.46, *p* = 0.026) after adjustment for age and body mass index. Conclusions: Lower PPAT-ADC values may be associated with higher biopsy ISUP grade group PCa and a higher percentage of PBx-positive cores. Higher-level studies are needed to confirm these preliminary results.

## 1. Introduction

Prostate cancer (PCa) is the most commonly diagnosed cancer and the second leading cause of death from cancer in men [1]. A wide variety of exogenous/environmental factors have been discussed as being associated with the risk of developing PCa. Specifically, PCa is associated with different eating habits determined by geographic variations, possibly related to different genetic susceptibilities. Among these, family history, BRCA gene mutations, and metabolic syndrome (especially hypertension and obesity) have been associated with a high risk of developing PCa [2]. Additionally, dietary factors such as alcohol intake have also been associated with a higher risk of PCa and PCa-related mortality [2]. Preventive interventions at all levels represent the cornerstone of adherence to disease treatment and progression avoidance [3].

Screening for PCa remains one of the most controversial topics in the urologic literature, and it is currently not recommended in most countries worldwide. An overall survival benefit is still lacking, despite additional evidence suggesting a long-term benefit of prostate-specific antigen (PSA)-based screening in terms of reducing cancer-specific mortality [2]. Informed men requesting an early diagnosis, such as men aged >50 years (>45 years in men of African descent) or with a family history of PCa, and men harboring BRCA gene mutations, should be given a PSA test and should undergo digital rectal examination (DRE), as recommended by international guidelines [2].

PCa diagnosis relies on prostate biopsy (PBx) [4,5]. In the last few years, the use of multiparametric magnetic resonance imaging (mpMRI) has greatly increased, showing an important improvement in clinically significant PCa diagnosis when used to guide PBx [6]. In this context, Prostate Imaging—Reporting and Data System version 2 (PIRADS vs2) provides a standardized risk classification of the lesions detected with mpMRI from 1 to 5 in the identification of PCa [7]. 

In a mpMRI setting, the apparent diffusion coefficient (ADC) is a measure of the extent of water molecules’ diffusion within tissues. This value is calculated using automatic software applied to MRI with diffusion-weighted imaging (DWI) and displayed as a parametric map [8]. The measurement of ADC values is carried out by selecting a specific region of interest (ROI) on the relevant finding displayed on the map [9]. This value is expressed in units of mm^2^/s. There is no consensus regarding normal restriction values, but ADC values less than 1.0 to 1.1 × 10^−3^ mm^2^/s (or 1000–1100 × 10^−6^ mm^2^/s) are generally known as indicative of adult restriction. However, this depends on the organ and the pathology, and a low ADC value is correlated with high organ cellularity [10]. 

Many studies have identified a direct association between obesity and more aggressive PCa biology in terms of grade, stage, presence of metastasis, and PCa-related mortality [11]. This association could be due to systemic obesity-related effects that increase serum growth factors and pro-inflammatory cytokine levels [11]. A specific association between obesity and an increase of periprostatic adipose tissue (PPAT), defined as the adipose bed that surrounds the prostatic surface, has also been proposed, as well as an interaction between periprostatic fat and prostatic microenvironment that might have a specific role in PCa induction and progression [12]. 

Here, we present a preliminary report on the association between PPAT-ADC and PBx findings in a single-center population. 

## 2. Materials and Methods

### 2.1. Study Population

We identified 219 consecutive patients undergoing PBx for suspicion of PCa, between January 2020 and June 2022, from our institutional review board-approved PBx database (Department of Urology, “Vito Fazzi” Hospital, Lecce, Italy; institutional review board of approval number 151983/2022). 

Indications to perform biopsies were increased PSA levels, abnormal DRE and/or abnormal imaging of the prostate. Patients undergoing any medical, as well as any previous surgical prostate treatment, were excluded. Only patients who performed MRI and PBx at our institution were included. These selection criteria yielded 132 assessable patients. 

For each patient, personal information such as age, body mass index (BMI; kg/m^2^), comorbidities, and related medications were collected in addition to the last available PSA value data and PSA density, defined as the ratio between PSA and prostate volume, evaluated with transrectal ultrasound (TRUS). 

### 2.2. MRI Acquisition

**Figure 1:**
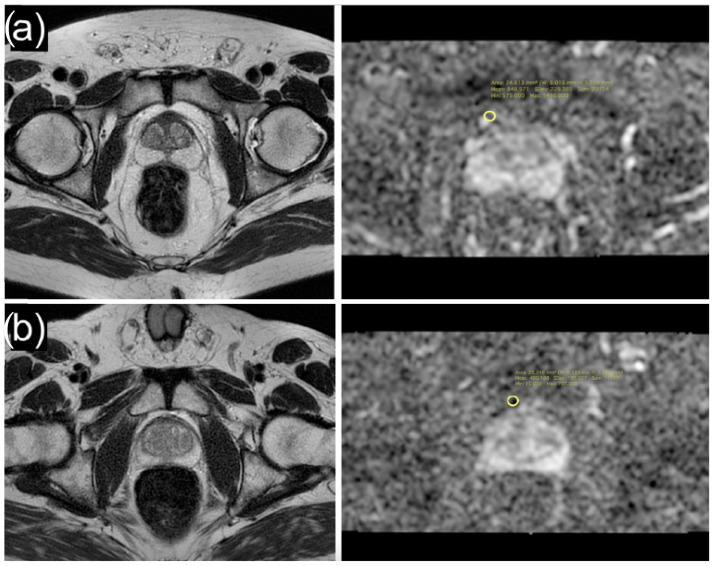
Axial T2-weighted image and ADC map of a 71-year-old patient with high ADC values (mean: 848.9 × 10^−6^ mm^2^/s) of periprostatic adipose tissue (**a**), and axial T2-weighted image and ADC map of a 64-year-old patient with low ADC values (mean: 405.1 × 10^−6^ mm^2^/s) of periprostatic adipose tissue (**b**).

MpMRI examinations were performed using a 1.5T scanner (Magnetom Signa Explorer GE healthcare, Waukesha, WI, USA) by positioning a dedicated 16-channel phased-array surface coil. Imaging sequences used for the study were T2-weighted (T2W) on axial, sagittal, and coronal planes, T1-weighted (T1W), and diffusion-weighted images (DWI) using b = 0 and b = 1400 (ADC map generated from b = 1400). Additional acquisitions included T1W with fat suppression dynamic contrast-enhancement (DCE) during the intravenous injection of 0.1 mL/kg gadobutrol (Gadovist/Gadavist, Bayer Pharma AG, Berlin, Germany) at 2 mL/s. This was followed by the infusion of 30 mL of saline solution at the same speed and T1W with fat suppression after contrast media injection on axial planes. All considered mpMRIs were reviewed and re-categorized according to PIRADS v.2.1 by dedicated radiologists with more than 10 years of experience with prostate MRI, who was blinded from the histology outcomes (FG, LG). ADC map correlated with T2W images were used for the acquisition of the ADC values. In particular, ROIs were positioned in the ADC map at the level of the periprostatic tissue compartment (Figure 1); the correlation with T2W images allowed the positioning of the ROIs in the precise anatomical site.

### 2.3. MRI/TRUS Fusion Biopsy

MRI-transrectal ultrasound fusion-guided prostate biopsies (MRI/TRUS-PBx) were performed by experienced urologists, with more than 200 MRI/TRUS-PBx by each operator, at a single institution. With the patient under local anesthesia and using an imaging fusion system (Virtual navigator Esaote^®^ System), at least two cores of target biopsy (TB) for each mpMRI-detected PIRADS 5 lesion were obtained followed by 18-cores systematic biopsy (SB, 7 per lobe in peripherical zone and 2 per lobe in transitional and central zone), all performed with an 18G needle. Each core was individually labeled and submitted to histology in separate containers. Biopsy specimens were analyzed by dedicated uro-pathologists and interpreted according to the International Society of Urological Pathology (ISUP) Grade Group (GG) [4].

### 2.4. Statistical Analyses

Descriptive statistics included frequencies and proportions for categorical variables. Median and interquartile ranges (IQR) were reported for continuously coded variables. Kruskal–Wallis rank sum test, Pearson’s Chi-squared test, and Fisher’s exact test were used to examine the statistical significance of differences in medians and proportions, respectively. Linear regression analyses tested the association between the PPAT-ADC and PCa clinical and pathological factors. These consisted of PSA, PSA density, prostate volume, PIRADS, ISUP score system, and the percentage of PBx-positive cores. Logistic regression analyses tested if PPAT-ADC was an independent predictor of intermediate-high risk PCa. All tests were two-sided with a level of significance set at *p* < 0.05. R software environment for statistical computing and graphics (version 4.1.2) was used for all analyses (R: the R Project for Statistical Computing. https://www.r-project.org, accessed on 14 July 2022).

## 3. Results

### 3.1. Descriptive Characteristics of the Study Population

**Figure 2:**
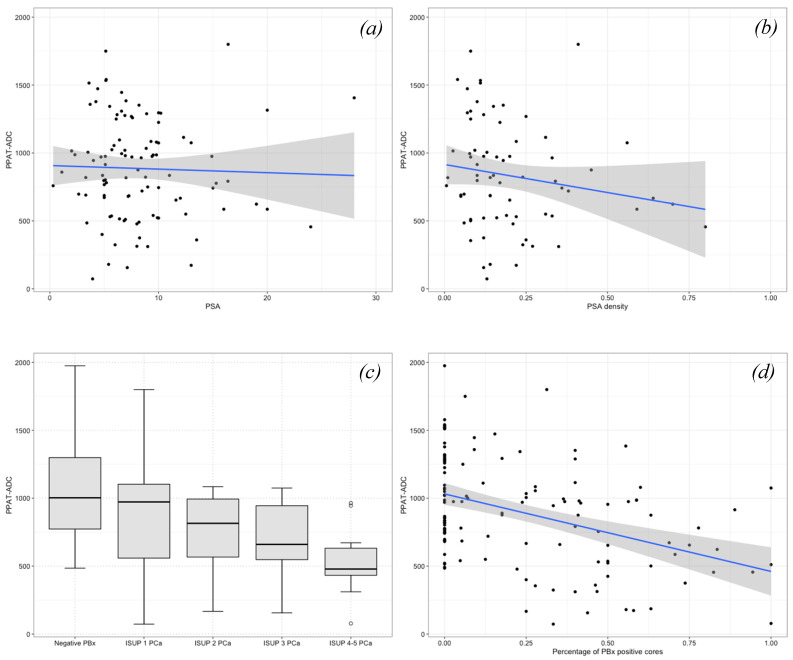
Scatter plots and box plots illustrating the relationship between periprostatic adipose tissue (PPAT)—apparent diffusion coefficient (ADC) values and (**a**) PSA, (**b**) PSA density, (**c**) ISUP grade group, and (**d**) percentage of prostate biopsy (PBx) positive cores. PPAT-ADC values decreased for higher PSA, PSA density, ISUP grade group, and percentage of PBx positive cores.

Overall, 132 patients were included (Table 1). Of these, 56 (42%) had negative PBx, 46 (35%) harbored ISUP 1 PCa, and 30 (23%) harbored ISUP > 1 PCa. Median PSA was 7.2 (IQR 5.1–10.0) ng/mL. Median PSA density increased with PCa aggressiveness (0.11 vs. 0.18 vs. 0.22 in respectively negative PBx, ISUP 1 PCa, and ISUP > 1 PCa; *p* < 0.001). At the mpMRI, a higher PIRADS score was associated with a higher ISUP grade group (*p* < 0.001). Compared to ISUP1 PCa, ISUP > 1 PCa patients had a higher median percentage of PBx positive cores (50 vs. 28, *p* < 0.001) and belonged to the intermediate-high D’Amico risk class. Overall, the median PPAT-ADC value was 876 (IQR 654–1112) × 10^−6^ mm^2^/s and decreased according to PCa aggressiveness (1003 vs. 972 vs. 656, in respectively negative PBx, ISUP 1 PCa and ISUP > 1 PCa patients; *p* < 0.001; see also Figure 2). 

### 3.2. Association between PPAT-ADC and PCa Clinical and Pathological Biopsy Features

As shown in Table 2, compared with patients with PPAT-ADC above the median (876 × 10^−6^ mm^2^/s), patients with PPAT-ADC up to the median had a higher rate of PIRADS 5 lesions at mpMRI (41% vs. 23%, *p* = 0.032), a higher percentage of PBx positive cores (25% vs. 6%, *p* = 0.049), and more frequently harbored ISUP > 1 Pca (50% vs. 28%, *p* = 0.048). At univariable linear regression analyses, the PSA (β = −3.17, 95%CI: −5.70, −0.64; *p* = 0.014), PSA density (β = −131.12, 95%CI: −242.63, −19.60; *p* = 0.022), PIRADS 5 (β = −180.54, 95%CI: −358.56, −2.52; *p* = 0.047), and percentage of PBx positive cores (β = −570.54, 95%CI: −789.64, −351.44; *p* < 0.001) were associated with lower PPAT-ADC values (Table 3, Figure 2). At multivariable logistic regression analyses, a PPAT-ADC up to the median was an independent predictor for intermediate-high risk PCa (odds ratio [OR]: 3.24, 95%CI: 1.17–9.46, *p* = 0.026), after adjustment for age and BMI (Table 4).

## 4. Discussion

Over the last 30 years, the prevalence of PCa has mirrored the increase in obesity and metabolic syndromes [13,14]. Several studies have evaluated the relationship between visceral obesity (estimated through BMI) and PCa incidence, features of aggressiveness, and outcomes. De Nunzio et al. found that obesity was associated with high-grade disease at the time of biopsy [15]. Kelly et al. suggested that increasing BMI during adulthood resulted in an increased risk of fatal PCa [16]. Freedland et al. found that higher BMI was associated with biochemical recurrence after radical prostatectomy [17]. In a recent meta-analysis, Gacci et al. demonstrated that the presence of metabolic syndrome predicted aggressive PCa and biochemical recurrence after treatment [18]. Further, it has been recently shown that increased BMI predicts the risk of multiple lymph node invasion after open and robot-assisted radical prostatectomy [19,20,21,22], as well as the risk of positive surgical margins and high-grade complications after surgery [23,24]. Several mechanisms have been proposed to explain the linkage between obesity and PCa. In overweight or obese patients, the risk of aggressive PCa is related to systemic effects such as dyslipidemia, increased serum concentrations of inflammatory factors like IL-6, IL-8, vascular endothelium growth factor, and leptin, as well as the deregulation of the insulin/insulin-growth factor 1 axis. All these factors can harm the prostatic microenvironment and cellular DNA. In addition, obesity has a pivotal role in altering the pituitary-testis axis in middle aged-men, causing decreased serum testosterone levels through an increase in peripheral androgen aromatization [11].

A specific association between visceral obesity and increased PPAT has also been hypothesized, as well as the interaction between periprostatic fat and prostatic microenvironment that might have a specific role in PCa induction and progression [12]. Specifically, it has been supposed that reciprocal interaction between adipocytes and tumor cells re-programmed adipocytes to a less differentiated status, referred to as cancer-associated adipocytes (CAAs) [12]. In turn, CAAs secrete several adipokines, cytokines, hormones, enzymes, and growth factors that may boost PCa cell growth and progression [12,25]. Fatty acids are also translocated from PPAT into PCa cells, increasing energy production. Obesity drives inflammation within the PPAT and modifies PPAT constituents, and transcriptomic, metabolic, and endocrine profiles, potentially augmenting their secretome [12]. These effects on the PPAT, taken together with the documented systemic effects of obesity, may support the associations between PPAT and increasing PCa aggressiveness [12,26,27].

In the last few years, the application of MRI in PCa detection has largely increased, with MRI as a valid tool to guide PBx [28]. In this contest, the PIRADS vs2 classification importantly increased the detection rate of clinically significant (CS) PCa at TB and reduced the diagnosis of non-clinically significant disease compared to SB [29]. For example, in the PRECISION study, the TB detection rate of CS PCa for PIRADS 5 lesions was 83%, and the detection rate of ISUP grade > 2 cancers was higher in men who underwent MRI-TB only with respect to the SB cohort [6]. The MRI-first study found a detection rate of 88% of CS PCa for PIRADS 5 lesions at TB [30]. A recent study demonstrated that men with negative MRI, PSA density ≤ 0.15 ng/mL^2^, and prior negative biopsy might safely avoid re-biopsy [31]. Considering the combination of MRI lesions and serum markers in PCa diagnosis, a recent report showed a detection rate in CS PCa detection for TB of 81% in PIRADS 5 patients. When the authors focused on PSA density > 0.15ng/mL^2^ sub-population, TB had a detection rate of 91%, and only three CS PCa patients were missed if SB was omitted [32]. Similarly, Lee et al. found in the same patients’ category that only 4 (5%) patients with any cancer and 6 (8%) patients with CS PCa would be missed if SB was omitted [33].

Due to this evidence, in the present study, we aimed to investigate the relationship between PPAT evaluated at pre-biopsy MRI during the ADC phase and PCa aggressiveness. We included 132 PBx patients with available MRI data, and among these, 76 patients had PCa (Table 1). Overall, the median PPAT-ADC value was 876 and decreased according to the increasing ISUP group (Table 2, Figure 2). In this cohort, lower PPAT-ADC values were associated with higher PSA, PSA density, a higher percentage of PBx positive cores, and PIRADS 5 (*p* < 0.05 in all cases; Table 3, Figure 2). Interestingly, PPAT-ADC inferior to the median was an independent predictor of the risk of harboring intermediate-high risk PCa at the biopsy after adjustment for the available covariates (OR: 3.24, *p* = 0.026; Table 4).

Several studies have previously investigated the relationship between PPAT area, volume, ratio (PPAT volume/prostate volume), density, or thickness, and PCa clinical features, with controversial results. Van Roermund et al. showed that total PPAT area and density measured on computed tomography (CT) were associated with high-grade PCa in a large cohort of patients treated with radiation therapy or brachytherapy [34]. Allot et al. reported no statistically significant association between PPAT area (CT evaluated) and risk of PCa aggressiveness in 308 patients submitted to radiation therapy; however, visceral obesity is associated with increased aggressive PC risk, particularly among black men [35]. Other studies relied on mpMRI findings for PPAT measurement. Tan et al. demonstrated that PPAT volume and PPAT ratio were statistically significantly associated with higher Gleason scores [36], while Zhang et al. proved that PPAT area was associated with a higher PCa stage and grade [37]. However, neither group of investigators relied on PPAT-ADC as a predictor of PCa aggressiveness. A study using high-resolution diffusion NMR Spectroscopy performed on rats fed chow and a high-fat diet showed significantly lower ADC values of white adipose tissue in rats fed with a high-fat diet. This study also demonstrated a significant increase in adipocyte size in rats fed high-fat diets due to large intracellular lipid accumulation [38]. This could be radiologically explained by increased saturation, decreased unsaturation, and increased mean chain length. ADC of fat molecules decreased with increases in mean chain length [39]. The reduced unsaturation of lipids makes them stiffer and more viscous. These properties probably cause a slowdown of diffusion [38].

Taken together, in the present study, we showed that patients with lower PPAT-ADC values recorded at pre-biopsy mpMRI might exhibit the most aggressive PCa profile at fusion-PBx in terms of PSA, PSA density, PIRADS classification, and percentage of PBx positive cores. Additionally, PPAT-ADC values up to the median were independently associated with the risk of developed intermediate-high risk PCa. Despite its novelty, the current study has limitations. These findings come from a retrospective cohort of patients who underwent fusion PBx in a high-volume center. However, the population was highly selected, and our expert uro-radiologists reviewed all images. This study might represent a starting point for prospective studies with a larger population that could investigate the role of PPAT-ADC in PCa aggressiveness prediction. If our findings are confirmed in higher-level studies, PPAT-ADC may be included in the MRI-pre biopsy setting to better manage PCa patients.

## 5. Conclusions

MRI-derived ADC of peri-prostatic adipose tissue may predict PCa features of aggressiveness. Specifically, lower PPAT-ADC values may be associated with a higher biopsy ISUP grade group and a higher percentage of PBx-positive cores. Higher-level studies are needed to confirm these preliminary results.

## Figures and Tables

**Table 1:** Descriptive statistics of 132 patients who underwent prostate biopsy (PBx) for suspicion of clinical prostate cancer (PCa) with available multiparametric-magnetic resonance imaging (mp-MRI) data.

Characteristic	Overall n = 132 ^1^	Negative PBx n = 56 (42%) ^1^	ISUP Grade Group 1 PCa n = 46 (35%) ^1^	ISUP Grade Group > 1 PCa n = 30 (23%) ^1^	*p*-Value ^2^
**Age (years)**	72 (65, 76)	68 (61, 75)	72 (66, 76)	74 (71, 78)	**0.002**
**BMI (Kg/m^2^)**	26.2 (23.9, 28.0)	26.4 (24.3, 29.0)	25.9 (23.9, 27.4)	25.4 (23.9, 28.5)	0.4
**Positive DRE**	57 (44%)	14 (25%)	22 (50%)	21 (70%)	**<0.001**
**PSA (ng/mL)**	7.2 (5.1, 10.0)	7.0 (5.0, 9.9)	7.0 (5.3, 10.0)	8.4 (6.0, 12.0)	0.2
**Prostate volume (ml)**	45 (33, 68)	58 (43, 84)	38 (31, 53)	42 (26, 60)	**<0.001**
**PSA density (ng/mL^2^)**	0.15 (0.09, 0.25)	0.11 (0.06, 0.15)	0.18 (0.08, 0.28)	0.22 (0.12, 0.50)	**<0.001**
**Number of lesions**					0.9
1	74 (56%)	32 (57%)	25 (54%)	17 (57%)	
>1	58 (44%)	24 (43%)	21 (46%)	13 (43%)	
**PIRADS**					**<0.001**
3	33 (25%)	27 (49%)	3 (6%)	3 (10%)	
4	58 (44%)	18 (33%)	32 (70%)	8 (27%)	
5	40 (31%)	10 (18%)	11 (24%)	19 (63%)	
**Periprostatic adipose tissue ADC**	876 (654, 1112)	1003 (773, 1299)	972 (559, 1103)	656 (455, 952)	**<0.001**
**Percentage of positive core**	9 (0, 41)	-	28 (13, 47)	50 (34, 72)	**<0.001**
**D’Amico risk group**					**<0.001**
Low	36 (48%)	-	36 (82%)	0 (0%)	
Intermediate	28 (38%)	-	8 (18%)	20 (67%)	
High	10 (14%)	-	0 (0%)	10 (33%)	

^1^ Median (IQR); n (%). ^2^ Kruskal–Wallis rank sum test; Pearson’s Chi-square test; Fisher’s exact test; *p*-values in bold are indicative of statistical significance (<0.05). Abbreviations: ISUP, International Society of Urological Pathology; BMI, Body Mass Index; DRE, digital rectal examination; PSA, prostate-specific antigen; PIRADS, prostate imaging reporting & data system; ADC, apparent diffusion coefficient.

**Table 2:** Descriptive statistics of 132 patients who underwent prostate biopsy (PBx) for suspicion of clinical prostate cancer (PCa) with available multiparametric-magnetic resonance imaging (mp-MRI) data, stratified according to median peri-prostatic adipose tissue (PPAT)—apparent diffusion coefficient (ADC): up to vs. above the median.

Characteristic	PPAT-ADC ≤ 876 n = 66 (50%) ^1^	PPAT-ADC > 876 n = 66 (50%) ^1^	*p*-Value ^2^
**Age (years)**	72 (62, 77)	72 (65, 76)	0.9
**BMI (Kg/m^2^)**	26.00 (24.20, 27.70)	26.20 (23.90, 28.33)	0.6
**Positive DRE**	33 (51%)	24 (37%)	0.11
**PSA (ng/mL)**	8.0 (5.2, 11.8)	6.9 (5.2, 9.5)	0.3
**Prostate volume (mL)**	41 (31, 66)	45 (36, 70)	0.3
**PSA density (ng/mL^2^)**	0.18 (0.11, 0.33)	0.12 (0.08, 0.18)	0.053
**PIRADS**			**0.032**
3	15 (23%)	18 (25%)	
4	24 (36%)	34 (52%)	
5	27 (41%)	13 (23%)	
**ISUP grade group**			**0.048**
1	20 (50%)	26 (72%)	
>1	20 (50%)	18 (28%)	
**Percentage of positive core**	0.25 (0.00, 0.50)	0.06 (0.00, 0.30)	**0.049**

^1^ Median (IQR); n (%) ^2^ Wilcoxon rank sum test; Pearson’s Chi-square test; *p*-values in bold are indicative of statistical significance (<0.05). Abbreviations: BMI, body mass index; DRE, digital rectal examination; PSA, prostate-specific antigen; PIRADS, prostate imaging reporting & data system; ISUP, International Society of Urological Pathology.

**Table 3:** Univariable linear regression analyses testing the association between determinant of prostate cancer aggressiveness and peri-prostatic adipose tissue (PPAT)—apparent diffusion coefficient (ADC) values.

Characteristic	Beta	95% CI ^1^	*p*-Value
**PSA**	−3.17	−5.70, −0.64	**0.014**
**PSA density**	−131.12	−242.63, −19.60	**0.022**
**Prostate volume**	1.01	−1.35, 3.55	0.4
**PIRADS**			
3	Ref	-	
4	−49.18	−214.24, 115.88	0.6
5	−180.54	−358.56, −2.52	**0.047**
**ISUP grade group**			
Negative PBx	Ref	-	
1	−158.79	−300.36, −17.22	**0.028**
2	−294.68	−538.92, −50.43	**0.018**
3	−351.28	−595.52, −107.03	**0.005**
4–5	−506.08	−750.32, −261.83	**<0.001**
**Percentage positive cores**	−570.54	−789.64, −351.44	**<0.001**

^1^ CI = Confidence Interval; *p*-values in bold are indicative of statistical significance (<0.05). Abbreviations: PSA, prostate-specific antigen; PIRADS, prostate imaging reporting & data system; ISUP, International Society of Urological Pathology; PBx. Prostate biopsy.

**Table 4:** Logistic regression analyses tested the association between peri-prostatic adipose tissue (PPAT)—apparent diffusion coefficient (ADC) and intermediate-high risk prostate cancer according to D’Amico classification.

	Univariable		Multivariable	
	OR (95% CI) ^1^	*p*-Value	OR (95% CI) ^1^	*p*-Value
**PPAT-ADC**				
>876	Ref	—	Ref	—
≤876	3.02 (1.19, 7.99)	**0.022**	3.24 (1.17, 9.46)	**0.026**
**Age (years)**	1.05 (0.99, 1.13)	0.1	1.04 (0.97, 1.12)	0.3
**BMI (Kg/m^2^)**	1.02 (0.87, 1.21)	0.8	1.03 (0.87, 1.24)	0.7

^1^ OR = Odds Ratio, CI = Confidence Interval; *p*-values in bold are indicative of statistical significance (<0.05).

## Data Availability

The data presented in this study are available on request from the corresponding author. The data are not publicly available.
